# General lifestyle factors explain young athletes’ mental health more than perceived coach autonomy support: a cross-sectional study on basketball players and gymnasts aged 10–22

**DOI:** 10.1136/bmjsem-2023-001648

**Published:** 2023-08-23

**Authors:** Carolina Lundqvist, Anna Asratian, Örjan Dahlström

**Affiliations:** 1 Department of Behavioral Sciences and Learning, Linköping University, Linkoping, Sweden; 2 Athletics Research Center, Department of Health, Medicine and Caring Sciences, Linköping University, Linkoping, Sweden; 3 Department of Biomedical and Clinical Sciences, Linköping University, Linkoping, Sweden

**Keywords:** health promotion, sleep, sports medicine, stress, well-being

## Abstract

**Objectives:**

This study described differences in lifestyle factors (sleeping problems/fatigue, pressure/activation), perceived coach autonomy support and indicators of mental health (well-being and poor general mental health) across various age groups (children ≤12 years, youths 13–15 years, junior to senior ≥16 years) and sports (basketball and gymnastics). Second, the relationships between lifestyle factors and mental health indicators were explored, hypothesising that the relationships would be mediated by perceived coach autonomy support.

**Methods:**

A cross-sectional study design was implemented by using an online survey which assessed lifestyle and environmental factors as well as mental health indicators. Participants were recruited through sports clubs in basketball and gymnastics. A total of 209 athletes (77 basketball players and 132 gymnasts) in the age range of 10–22 (median=13) years volunteered to complete the survey.

**Results:**

Separate two-way analyses of variance showed significant main effects for age group on sleeping problems/fatigue, sleep quantity, pressure/activation, well-being and poor general mental health, with higher scores reported for older age groups of athletes. Path analysis displayed sleeping problems/fatigue and pressure/activation to significantly affect decreased well-being and poor general mental health; however, the relationships were not mediated by perceived coach autonomy support.

**Conclusion:**

Lifestyle factors play a prominent role in mental health outcomes. Researchers studying athlete mental health should consider both general lifestyle and sports-related factors, considering developmental phases in the young athlete’s sporting context and overall life.

**WHAT IS ALREADY KNOWN ON THIS TOPIC**

- The WHO estimates that 14% of young people aged 10–19 experience mental health problems and mental disorders. Mental health is multifaceted and can be affected by factors like biology, resilience, sleep, environment, diet, stress and physical activity.
- Participation in organised sports has the potential to be protective of mental health. Still, contemporary research shows a weak negative relationship between young athletes’ sports involvement and mental health problems.
- Researchers suggest coach autonomy support as an environmental precursor for athletes’ motivation, continued sports participation and well-being. Team sports participation seems particularly beneficial for young athletes’ positive mental health.

**WHAT THIS STUDY ADDS**

- Age is essential to consider in research on athletes’ mental health. Older athletes (aged 13–15 and ≥16) reported greater sleeping problems/fatigue, more pressure/activation, lower well-being and poorer general mental health than younger athletes.
- General lifestyle factors (sleeping problems/fatigue, pressure/activation) directly correlate with decreased well-being and poorer general mental health. The relationships were not mediated by perceived coach autonomy support.

**HOW THIS STUDY MIGHT AFFECT RESEARCH, PRACTICE OR POLICY**

- The findings stress the importance of researchers and practitioners targeting athlete mental health from a holistic view. The full spectrum of general lifestyle factors and sports-related precursors for athlete mental health variations in various ages and developmental phases should be considered.
- Universal mental health education programmes in sports settings should be prioritised to increase young athletes’ knowledge of self-care strategies and to promote healthy personal and athletic development and performance.

## Introduction

Globally, the WHO estimates that 14% of young people aged 10–19 experience mental health problems and mental disorders, which can lead to a health-related disability during adolescence and long-lasting consequences.^1^ Children and adolescents commonly report stress, pressures in life and inadequate sleep, which can adversely affect mental health.^2 3^ Mental health is multifaceted and affected by many factors like biology, resilience, sleep, environment, diet, stress and physical activity.^4–10^ Physical activity can stabilise mood, increase well-being and mental health by affecting brain circuits important for cognitive function (eg, attention, working memory capacities, cognitive control).^11–14^ Maintaining good mental health also involves, for example, mental health literacy, social skills, emotional regulation, functional stress management and a lifestyle with healthy behaviours.^15 16^

Emerging evidence shows that participation in organised sports can protect adolescents’ mental health and help develop positive health behaviours.^16 17^ Panza *et al*^18^ observed a weak yet significant negative association between sports involvement and self-reported anxiety and depression among athletes aged 10–20. Likewise, Rodriguez-Ayllon *et al*^19^ found a weak but significant relationship between physical activity and positive mental health among adolescents but no significant relationship for children.

Participation in team sports appears to be more beneficial to mental health than individual sports.^14 17 20^ For example, Gorham *et al*^14^ studied a nationwide sample of children aged 9–11 and found that participation in team sports, but not individual sports, was protective against depression. Similarly, a study on a representative American sample of children and adolescents aged 9–13 demonstrated that individual sports participation was linked to more mental health problems. In contrast, team sport participation was associated with fewer mental health concerns.^21^ Furthermore, large-scale cohort studies focusing on high school and senior high school students indicate team sport participation to be more related to positive mental health than individual sports during adolescence and early adulthood.^22 23^

Social support perceived within a team can potentially help athletes protect their mental health.^14^ Some individual sports, such as gymnastics, have an early peak performance age which may expose athletes to high training loads, psychosocial demands and competitive stress at a younger age than many team sports. While these circumstances can foster resilience, they can also threaten mental health and decrease well-being.^24^ Substantial research suggests coach autonomy support to be an essential environmental precursor for athletes’ motivation, sports involvement and well-being regardless of sports type and culture.^25^

Studies that investigated lifestyle and sports-related factors across age groups of athletes and individual and team sports are still sparse in the literature. Moreover, attention in research is needed to explore potential mediating psychosocial variables in the relationship between organised sports and athletes’ mental health.^16^ The first objective of this study was, therefore, to describe lifestyle factors (sleeping problems/fatigue, pressure/activation), one sports environmental factor (perceived coach autonomy support) and indicators of mental health (well-being and poor general mental health) across age categories of athletes (children ≤12 years, youths 13–15 years, junior to senior ≥16 years) and sports (basketball and gymnastics). Second, we aimed to explore the pathway through which lifestyle factors relate to mental health indicators, hypothesising that the association would be mediated by perceived coach autonomy support.

## Methods

### Study design

A cross-sectional study design was implemented using an online survey. This study was part of a larger data-collection in the project ‘Young people should feel good: Mental health through sports’. The Strengthening the Reporting of Observational Studies in Epidemiology guidelines are followed.

### Settings and participants

Participants were recruited through one team sport (basketball) and one individual sport (gymnastics) club in Sweden, which had approximately 170 and 440 members, respectively, eligible for this study. All athletes within the targeted age range of 10–25 years, actively participating in a recreational to national elite-level training group or team within these sports clubs, were invited and supplied with information about the study.

### Patient and public involvement statement

This study was performed in collaboration with sports clubs that wanted an increased understanding of mental health promotion and prevention. The sports clubs were involved in designing the survey and assisted during distribution.

### Data collection

The survey was completed anonymously on an electronic device (eg, tablet, smartphone) during practice, and an adult was present to answer any questions. No personal or sports information (eg, playing position, sports merits) that could reveal the athletes' identity was collected.

### Measures

Demographic information collected related to age, self-assigned gender, years athletes had practised the sport and number of other sports currently practised.

The 11-item Garmy Sleep Questionnaire (GSQ)^26^ originally measured sleep habits in school-aged youths. In this study, three of the items about sleeping problems and fatigue were used: ‘How often I have difficulties in falling asleep’, ‘How often I feel tired in school’ and ‘I find it difficult to wake up in the morning’. The first and second author (CL and AA) also developed an additional sports-related question for this study: ‘How often I feel tired during basketball/gymnastics practice’. These items were rated on a 4-point scale (from 1 ‘never’ to 4 ‘always’). A higher score indicates greater sleeping problems and fatigue. Cronbach’s alpha for these four questions was 0.66. The survey also included two questions from the GSQ about sleep quantity nights before schooldays and when being free the next day. These two scores were combined into a total score of average sleep quantity (hours).

Athletes’ perceived pressure and activation were measured by the 11-item Pressure and Activation Stress Scale (PASS),^27^ showing acceptable psychometric properties in previous studies.^28 29^ The scale assesses ‘pressure’ (eg, feeling demands/being under pressure, not having time enough, perceiving multiple obligations) and ‘activation’ (difficulties relaxing, going on at high speed during days). For this study, an additional item was included (‘I feel pressure from the demands of basketball/gymnastics’), resulting in 12 items combined into a global score. The items were rated on a 5-point scale (from 1 ‘never’ to 5 ‘always’). Cronbach’s alpha in this study was 0.89.

The impact of coaching behaviour on the athlete was assessed by the Sport Climate Questionnaire (SCQ). The six items from the SCQ are based on the self-determination theory measuring the coach’s autonomy support (eg, ‘I feel that my coach provides me choices and options’) and were originally adapted from the Health-Care Climate Questionnaire.^30 31^ Responses are scored on a 7-point rating scale (from 1 ‘strongly disagree’ to 7 ‘strongly agree’). A higher score indicates a higher perceived coach autonomy support. Previous studies have shown adequate internal consistency,^31^ and Cronbach’s alpha in this study was 0.90.

Well-being was assessed by the five-item WHO Well-being Index (WHO-5 WBI), validated in multiple studies.^32^ The WHO-5 WBI items assess overall well-being (eg, ‘I have felt cheerful and in good spirits’) answered by a 6-point scale ranging (from 5 ‘all the time’ to 0 ‘never’). The total raw score was used, ranging from 0 to 25. A high score indicates a good well-being. Cronbach’s alpha in this study was 0.84.

The General Health Questionnaire (GHQ-12)^33^ assesses general mental health through 12 items (eg, ‘loss of sleep over worry’, ‘able to concentrate’) scored on a 4-point scale. Positive items were scored from 0 (‘much more than usual’) to 3 (‘not at all’), and negative items from 3 (‘much more than usual’) to 0 (‘not at all’). Several scoring procedures are used in the literature,^34^ and because we did not study psychiatric morbidity in need of cut-off scores, the Likert method for coding was chosen. A high score of GHQ-12 indicates poor general mental health. Cronbach’s alpha in this study was 0.79.

### Statistical analyses

**Figure 1:**
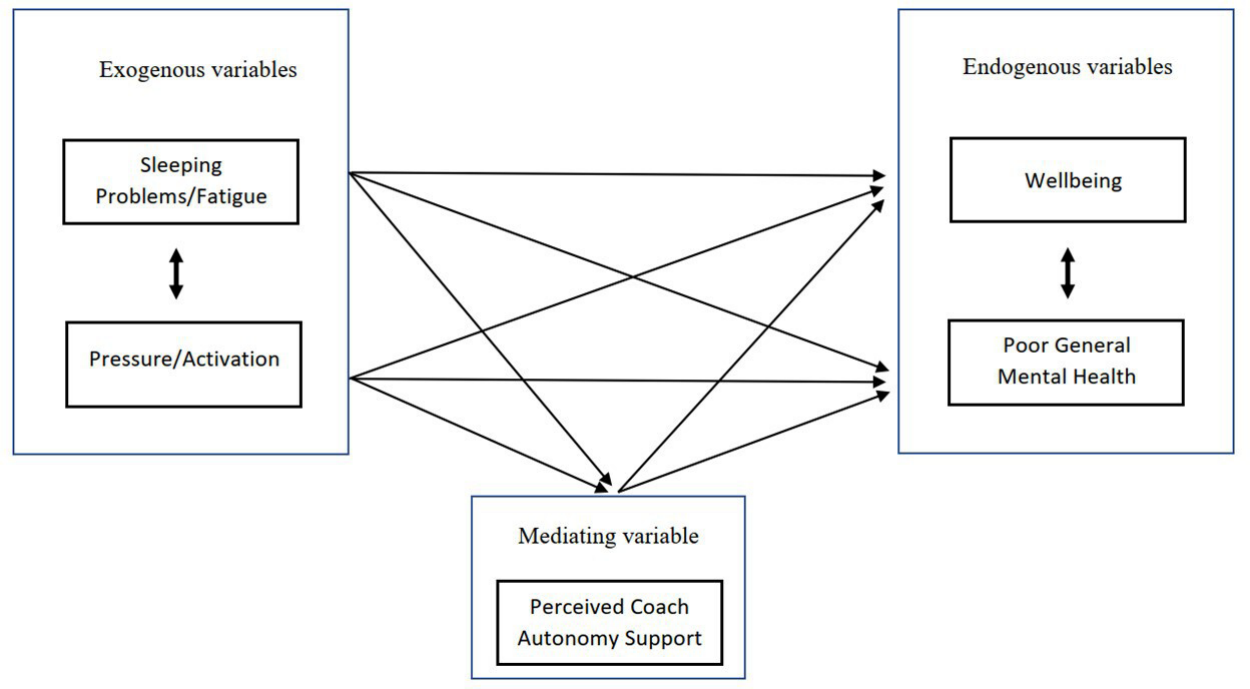
Hypothesised model displaying exogenous, endogenous and mediating variables included in the path analysis.

First, separate two-way analyses of variance with post hoc Bonferroni tests were used to investigate the interaction of sports and age group on scores of sleeping problems/fatigue (GSQ), sleep quantity (GSQ), well-being (WHO-5 WBI), poor general mental health (GHQ-12), perceived coach autonomy support (SCQ) as well as pressure/activation (PASS). Missing data were listwise deleted. Participants were classified into age groups based on the structure of the sporting and school system in Sweden; child sports ≤12 years, youth sports 13–15 years and junior to senior sports ≥16 years. Second, we explored our hypothesised model (figure 1), where well-being and poor general mental health were a priori specified as correlated endogenous variables, sleeping problems/fatigue and pressure/activation as correlated exogenous variables and perceived coach autonomy support as a mediating variable. This was done by examination of pairwise correlations followed by path analysis. Descriptive and univariate statistics were analysed using SPSS Statistical Package V.29, while path analysis was performed in MPLUS V.8 using maximum likelihood robust (MLR) estimation.

## Results

### Descriptives and comparisons between sports and age groups

**Figure 2:**
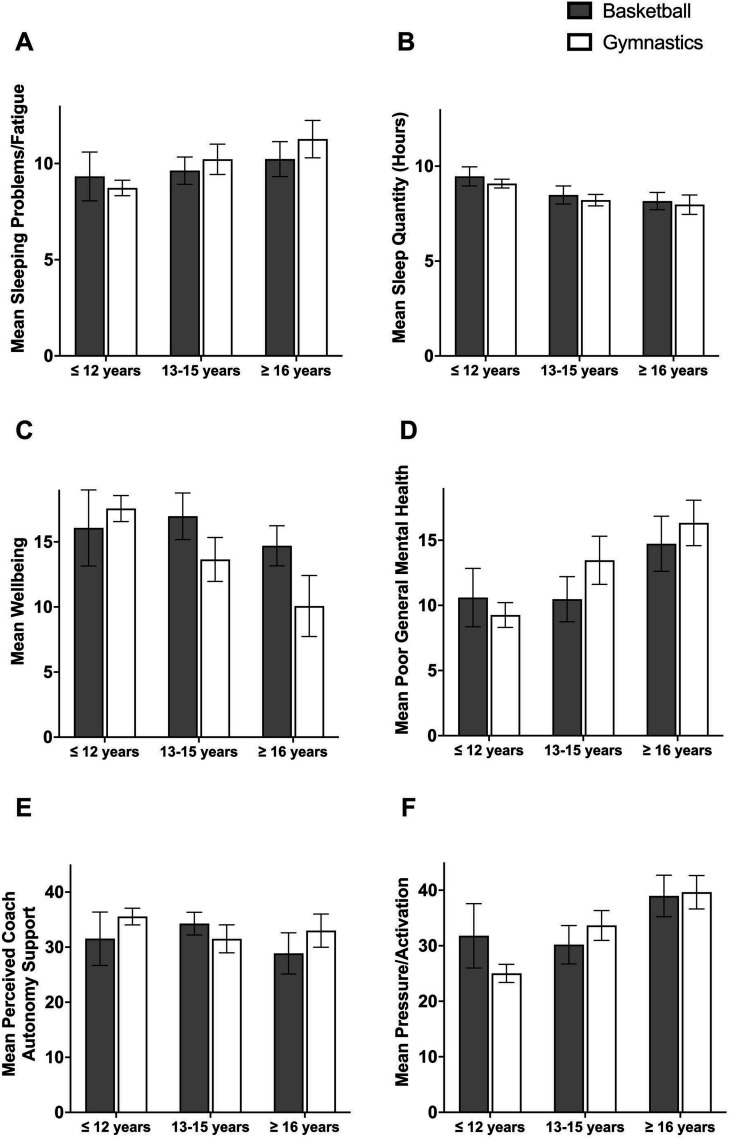
Comparison of mental health indicators in the age groups ≤12 years, 13–15 years and ≥16 years in basketball and gymnasts; (A) sleeping problems/fatigue, (B) sleep quantity, (C) well-being, (D) poor general mental health, (E) perceived coach autonomy support and (F) pressure/activation.

A total of 209 athletes (basketball players n=77 and gymnasts n=132) volunteered to complete the survey, corresponding to a response rate of 45% and 30% in each sport. In table 1, descriptives of gender distribution, the number of years participants had practised their sports and the number of other current sports are reported for age groups and sports. Shown are also the mean values of all assessments. The age range of the participants in the sample was 10–22 years (median=13 years). The data were approximately normally distributed (table 2), and no multivariate outliers were detected by use of Mahalanobis distance (χ^2^(5)≤15.09, p>0.001). The effect of age group and sports on self-reported mental health indicators and perceived coach autonomy support are shown in figure 2.

**Table 1:** Descriptives of participants and mean values of assessments

Sport	Age group	Gender	Years in sport	No of other sports	Sleeping problems/fatigue (GSQ)		Sleep quantity (GSQ)		Well-being (WHO-5 WBI)		Poor general mental health (GHQ-12)		Coach autonomy support (SCQ)		Pressure/activation (PASS)	
		Female/male/ other/not disclosed	Mean (SD)	Mean (SD)	n	Mean (SD)	n	Mean (SD)	n	Mean (SD)	n	Mean (SD)	n	Mean (SD)	n	Mean (SD)
Basketball	≤12	15/0/0/0	3.50 (1.56)	1.60 (.91)	15	9.33 (2.29)	14	9.46 (.87)	15	16.07 (5.27)	15	10.60 (4.05)	15	31.53 (8.75)	14	31.78 (10.04)
	13–15	12/17/0/1	4.90 (2.26)	1.33 (.71)	30	9.63 (1.90)	30	8.48 (1.28)	30	16.97 (4.79)	30	10.47 (4.63)	30	34.27 (5.52)	26	30.19 (8.55)
	≥16	16/14/0/0	8.03 (2.99)	1.30 (.79)	30	10.23 (2.43)	29	8.15 (1.20)	30	14.70 (4.12)	30	14.73 (5.67)	30	28.87 (10.01)	28	38.96 (9.66)
	Total	43/31/0/1	5.91 (3.10)	1.37 (.79)	75	9.81 (2.20)	73	8.54 (1.26)	75	15.88 (4.68)	75	12.20 (5.33)	75	31.56 (8.46)	68	34.13 (10.07)
Gymnastics	≤12	67/8/1/2	5.13 (3.47)	1.71 (.69)	78	8.73 (1.78)	76	9.08 (1.00)	78	17.56 (4.42)	78	9.26 (4.22)	78	35.55 (6.71)	59	25.02 (6.29)
	13–15	30/7/0/0	6.62 (3.22)	1.65 (.68)	37	10.22 (2.36)	34	8.21 (.86)	37	13.65 (5.06)	37	13.46 (5.55)	37	31.51 (7.59)	34	33.65 (7.72)
	≥16	15/0/0/0	10.27 (2.87)	1.27 (.46)	15	11.27 (1.75)	15	7.97 (.92)	15	10.07 (4.23)	15	16.33 (3.15)	15	33.00 (5.45)	14	39.64 (5.23)
	Total	112/15/1/2	6.16 (3.69)	1.64 (.67)	130	9.45 (2.15)	125	8.71 (1.06)	130	15.58 (5.27)	130	11.27 (5.20)	130	34.11 (7.03)	107	29.67 (8.59)
Total	≤12	82/8/1/2	4.88 (3.30)	1.69 (.73)	93	8.83 (1.87)	90	9.14 (.98)	93	17.32 (4.57)	93	9.47 (4.20)	93	34.90 (7.18)	73	26.31 (7.57)
	13–15	42/24/0/1	5.85 (2.94)	1.51 (.50)	67	9.95 (2.17)	64	8.35 (1.08)	67	15.13 (5.17)	67	12.12 (5.33)	67	32.75 (6.83)	60	32.15 (8.20)
	≥16	31/14/0/0	8.78 (3.10)	1.29 (.70)	45	10.58 (2.26)	44	8.09 (1.11)	45	13.15 (4.67)	45	15.27 (4.99)	45	30.24 (8.91)	42	39.19 (8.38)
	Total	155/46/1/3	6.06 (3.47)	1.54 (.72)	205	9.58 (2.17)	198	8.65 (1.14)	205	15.69 (5.05)	205	11.61 (5.26)	205	33.18 (7.67)	175	31.40 (9.42)

GHQ-12, General Health Questionnaire; GSQ, Garmy Sleep Questionnaire; PASS, Pressure and Activation Stress Scale; SCQ, Sport Climate Questionnaire; WHO-5 WBI, WHO Well-being Index.

**Table 2:** Pearson product moment correlations (r)

Variable	2	3	4	5	6	Skewness	Kurtosis
	Pearson R						
1. Sleeping problems/fatigue (GSQ)	−0.26* (n=202)	−0.64* (n=209)	0.59* (n=209)	−0.24* (n=209)	0.52* (n=178)	0.30	−0.01
2. Sleep quantity (GSQ)	-	0.33* (n=202)	−0.36* (n=202)	0.11 (n=202)	−0.45* (n=172)	−0.51	0.87
3. Well-being (WHO-5 WBI)	-	-	−0.70* (n=209)	0.33* (n=209)	−0.63* (n=178)	−0.39	−0.27
4. Poor General Mental Health (GHQ-12)	-	-	-	−0.25* (n=209)	0.77* (n=178)	0.76	0.51
5. Coach Autonomy Support (SCQ)	-	-	-	-	−0.26* (n=178)	−1.040	0.58
6. Pressure/activation (PASS)	-	-	-	-	-	0.37	−0.60

*p<0.05.

.GHQ-12, General Health Questionnaire; GSQ, Garmy Sleep Questionnaire; PASS, Pressure and Activation Stress Scale; SCQ, Sport Climate Questionnaire; WHO-5 WBI, WHO Well-being Index.

For sleeping problems/fatigue, there was a significant main effect for age group (F(2,199)=7.91, p=<0.001, partial η^2^=0.07) but not for sports (F(1,199)=1.02, p=0.31). The age group of athletes being ≤12 years had significantly lower scores than athletes being 13–15 years (p=0.002) and ≥16 years (p<0.001). There was no significant interaction between sports and age group (F(2,199)=2.02, p=0.14). Similar results were revealed for sleep quantity with a significant main effect for age group (F(2,192)=16.85, p=<0.001, partial η^2^=0.15) but not for sports (F(1,192)=2.67, p=0.10). The age group of athletes being ≤12 reported more hours of sleep than the two older age groups (p<0.001). No significant interaction was found between sports and age group on sleep quantity (F(2,192)=0.10, p=0.91).

For well-being, there were significant main effects for sports (F(1,199)=8.18, p=0.005, partial η^2^=0.04, basketball players showed higher scores) and age groups (F(2,199)=10.50, p<0.001, partial η^2^=0.10, age group ≤12 years old showed higher scores than the other age groups, p<0.05). There was also a significant interaction between sports and age groups (F(2,199)=5.92, p=0.003, partial η^2^=0.06). Analysis of simple main effects showed significantly higher scores for basketball than gymnastics in the two oldest age groups (13–15 years: p=0.004 and >16 years: p=0.002). At the same time, there were no such differences in the age group ≤12 years (p=0.25).

For poor general mental health, there was a significant main effect for age group (F(2,199)=16.01, p<0.001, partial η^2^=0.14) but not for sports (F(1,199)=1.99, p=0.16). Older athletes in all age groups reported higher scores (all p:s<0.01). There was also a significant interaction of sports and age group (F(2,199)=3.08, p=0.048, partial η^2^=0.03). Simple main effects showed that basketball players being 13–15 years old displayed lower scores than gymnasts in the same age group (p=0.01). At the same time, there were no such differences at age groups ≤12 years (p=0.31) or ≥16 years (p=0.28).

For perceived coach autonomy support, there were no significant main effects either for age group (F(2,199)=1.49, p=0.23) nor for sports (F(2,199)=2.24, p=0.14). Still, results showed a significant interaction between sports and age category (F(2,199)=4.09, p=0.02, partial η^2^=0.04). Analysis of simple main effects showed that in gymnastics there were higher scores for athletes ≤12 compared with those being 13–15 years (p=0.02), while for basketball athletes being 13–15 years scored higher compared with those being ≥16 years (p=0.02).

For pressure/activation, there was a significant main effect for age group (F(2,169)=20.56, p<0.001, partial η^2^=0.20) but not for sports (F(1,169)=0.43, p=0.51). Athletes ≤12 years had significantly lower scores than athletes aged 13–15 years (p=0.001) and ≥16 years (p<0.001), while athletes 13–15 years had significantly lower scores than those aged ≥16 years (p<0.001). There was also a significant interaction of sports and age group (F(2,169)=5.66, p=0.004, partial η^2^=0.06). Simple main effects showed that basketball players aged ≤12 years reported significantly higher pressure/activation than gymnasts in the same age group (p=0.004).

### Pairwise correlations and path analysis

Pearson product moment correlations (table 2) performed on the total sample showed expected directions and strengths. Sleeping problems/fatigue and pressure/activation were negatively correlated with well-being and positively correlated with poor general mental health, whereas sleep quantity showed correlations in the opposite direction. Perceived coach autonomy support was negatively related to sleeping problems/fatigue, but no significant relationship with sleep quantity was found. Perceived coach autonomy support was positively correlated with well-being and showed a negative relationship with poor general mental health.

**Figure 3:**
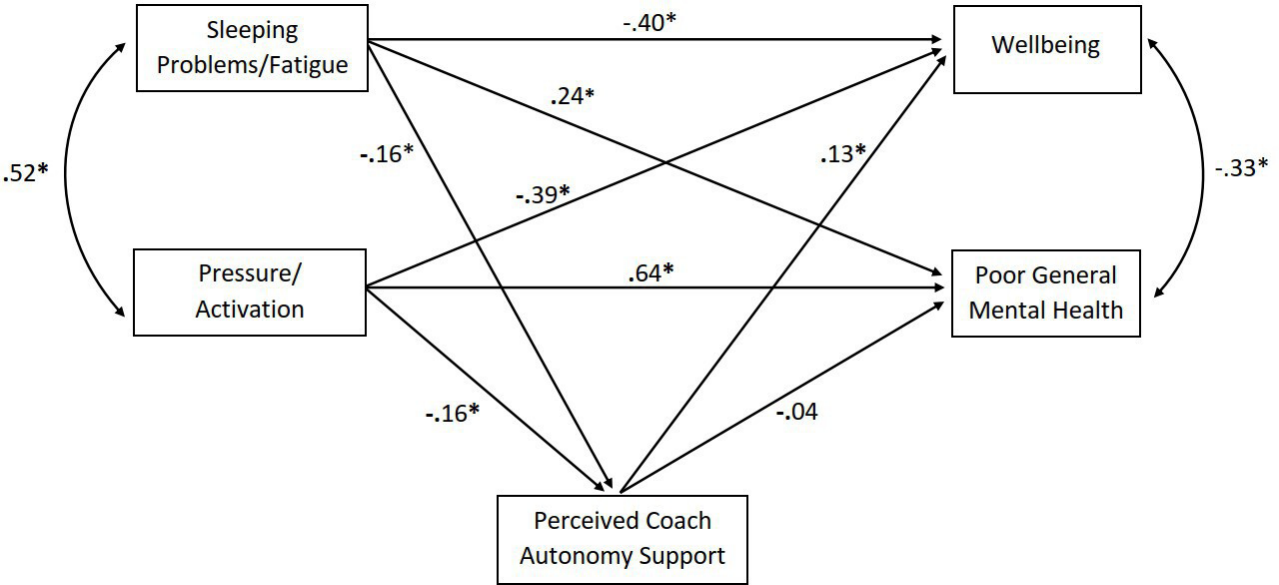
Standardised estimates from the path analysis. *p<0.05.

Results from the path analysis are shown in table 3 (direct, indirect and total effects) and in figure 3 (standardised coefficients). Sleeping problems/fatigue showed significant direct effects on decreased well-being and poor general mental health. Likewise, pressure/activation showed significant direct effects on decreased well-being and poor general mental health. No significant indirect effects were found, suggesting that the relationships were not mediated by perceived coach autonomy support.

**Table 3:** Standardised direct, indirect and total effects from the path analysis

Exogenous variables	Endogenous variables	Mediating variable	Direct effect	Indirect effect	Total effect
Sleeping problems/fatigue	Well-being	Perceived coach autonomy support	−0.40*	−0.02	−0.42*
Pressure/activation			−0.39*	−0.02	−0.41*
Sleeping problems/fatigue	Poor general mental health	Perceived coach autonomy support	0.24*	0.01	0.25*
Pressure/activation			0.64*	0.01	0.64*

*p<0.05.

## Discussion

This study investigated lifestyle factors, coach autonomy support and mental health indicators among young athletes in basketball and gymnastics. A strength of the study was the wide age range among participants, which enabled comparison across age groups of athletes. Results showed that older athletes in both basketball and gymnastics overall reported poorer well-being and general mental health than younger athletes. Moreover, general lifestyle factors in terms of sleeping problems/fatigue and perceived pressure/activation were strongly associated with lower scores of well-being and higher scores of poor general mental health. The hypothesised mediating effect of perceived coach autonomy support was not supported.

It is known that several common mental disorders (eg, depression, anxiety syndromes) have their peak onset age in the mid-teens to the mid-20s.^35^ Adolescence and early adulthood are vulnerable stages biologically, psychologically, and socially and the transition from youth to adulthood imposes an increased proportion of psychosocial stressors in the young person’s life.^36^ This life period also coincides with the age for junior-to-senior transition in sports, which can add additional sports-related stressors on adolescent athletes.^16^ In this study we found direct relationships between sleeping problems/fatigue, perceived pressure/activation and indicators of poor general mental health, with higher scores reported in older age groups. The results suggest that societal changes and general stressors in everyday life affect adolescent athletes, such as the general population. For example, in Sweden, an alarming trend of poor mental health (eg, stress-related symptoms, psychosomatic disorders, mood disorders) among young people is noticed in statistical reports, which can partly be related to changes in the school system and a harsher climate to enter the labour market.^37^ Researchers must recognise that young athletes, like the general population, face similar societal challenges and pressures that can affect their mental health, regardless of their involvement in sports. Overall, the results support the benefits of investigating athlete mental health holistically to identify both non-sports and sports-related precursors for athlete mental health variations in different ages.

### Clinical implications

While physical activity per se can protect mental health, the results in this study particularly suggest lifestyle factors among young athletes to play a prominent role in mental health outcomes. The study findings support previous research^38 39^ suggesting the benefits of implementing universal mental health education programmes in sports systems to enhance knowledge of mental health prevention and effective self-help strategies. Practitioners should recognise that young athletes may lack adequate self-care and stress-management skills to protect their mental health in sports and their broader life situations. The study findings also revealed that basketball players in the two oldest age groups reported higher well-being than gymnasts in the same ages. In addition, basketball players aged 13–15 years displayed lower scores of poor general mental health than gymnasts in the same age. These results partly support previous research indicating that team sports can protect mental health.^14 17 20–23^ However, the differences found across sports could also be a result of different peak performance ages in basketball and gymnastics, which can impact on training loads as well as psychosocial and competitive demands athletes face at various ages. Emerging evidence also indicates that adolescents’ continued team sports participation over time can mitigate experienced mental health challenges, enhance coping abilities and reduce stress levels later in young adulthood.^20^ These findings should be further explored in future research.

### Limitations

In this study, we adopted a cross-sectional design which limits a closer and more in-depth analysis of contextual factors within or outside sports (eg, family and peer support, socioeconomic factors, school demands, sports culture) that may influence participants reported mental health or health variations over time. Poor mental health can be followed by a decreased participation in physical activity,^40^ whereby athletes with exaggerated mental health problems could already have dropped out of sports and not be found in this study. Moreover, the data were collected from two different sports clubs in Sweden, limiting the findings’ generalisability. The study included a mixed sample with participants at different sporting levels (from recreational to national elite), and it is important to note that sports-related stressors generally increase with higher levels of competition. Future research should employ longitudinal designs with larger cohorts of young athletes followed over a longer period to explore the onset of mental health concerns and drop-outs and investigate precursors to mental health trajectory in sports more comprehensively. Moreover, future studies are encouraged to examine both non-sports and sports-related factors as potential risks and protective factors for mental health among young athletes.

In conclusion, general lifestyle factors (sleeping problems/fatigue, perceived pressure/activation) were strongly related to decreased well-being and poor general mental health among young athletes. Researchers studying athlete mental health should consider general lifestyle and sports-related factors, considering developmental phases in the sporting context and overall life.

## Data Availability

Data are available from the first author on reasonable request.
